# The Renin–Angiotensin System in Liver Disease

**DOI:** 10.3390/ijms25115807

**Published:** 2024-05-27

**Authors:** Mary S. McGrath, Brian J. Wentworth

**Affiliations:** 1 Department of Medicine, School of Medicine, University of Virginia, Charlottesville, VA 22903, USA; xua4yg@uvahealth.org; 2 Division of Gastroenterology & Hepatology, School of Medicine, University of Virginia, Charlottesville, VA 22903, USA

**Keywords:** angiotensin II, angiotensin 1–7, classical pathway, alternative pathway, cirrhosis

## Abstract

The renin–angiotensin system (RAS) is a complex homeostatic entity with multiorgan systemic and local effects. Traditionally, RAS works in conjunction with the kidney to control effective arterial circulation, systemic vascular resistance, and electrolyte balance. However, chronic hepatic injury and resulting splanchnic dilation may disrupt this delicate balance. The role of RAS in liver disease, however, is even more extensive, modulating hepatic fibrosis and portal hypertension. Recognition of an alternative RAS pathway in the past few decades has changed our understanding of RAS in liver disease, and the concept of opposing vs. “rebalanced” forces is an ongoing focus of research. Whether RAS inhibition is beneficial in patients with chronic liver disease appears to be context-dependent, but further study is needed to optimize clinical management and reduce organ-specific morbidity and mortality. This review presents the current understanding of RAS in liver disease, acknowledges areas of uncertainty, and describes potential areas of future investigation.

## 1. Introduction

The renin–angiotensin system (RAS) was first described in the late 19th century after discovery of a renally derived substance that modulated blood pressure [1]. However, its complexity and involvement in other systemic pathways were initially underappreciated. Over time, our understanding of RAS has evolved and it is now implicated in the pathogenesis of numerous acute and chronic conditions including COVID-19, renal dysfunction, diabetes mellitus, hypertension, and cardiac disease, including heart failure and myocardial infarction [2,3]. RAS has traditionally been considered to be upregulated in these conditions, promoting inflammation and vasoconstriction through angiotensin II (AngII) and its principal receptor, angiotensin type I receptor (AT1R) [4]. To counter these harmful effects, institution of hormonal blockade with various agents, including angiotensin converting enzyme inhibitors (ACEis) and angiotensin receptor blockers (ARBs), has been shown to improve clinical outcomes. This “classical” view of RAS, however, was challenged by the discovery of an alternative pathway with complex but often opposing physiologic actions, including anti-inflammatory and vasodilatory properties mediated through the vasoactive peptide angiotensin 1–7 (Ang1–7) [4]. In a paradigm shift, current scientific understanding now recognizes an imbalance between two variant but interdependent RAS pathways (henceforth referred to as the classical and alternative pathways) [5]. Further research has also demonstrated that the RAS system exerts endocrine, paracrine, and autocrine functions [6,7].

While there is a wealth of literature characterizing RAS in cardiovascular and renal disease, the role of RAS in liver disease is less well defined. Upregulation of RAS in decompensated cirrhosis to counteract splanchnic vasodilation is well appreciated. In contrast, the effects of RAS on hepatic fibrosis and the utility of hormonal blockade across the spectrum of liver disease are emerging areas of interest. The worldwide burden of liver disease is ever-growing, driven by high rates of alcohol abuse and metabolic-associated conditions, as well as improvements in life-sustaining therapies [8,9]. Its impact in the public health space is notable, as liver disease is the eleventh leading cause of death internationally and a major contributor to United States healthcare spending [10]. Thus, characterization of RAS in liver disease is important from both a clinical and public health perspective. Specifically, optimal usage of current RAS inhibitory therapies is important to improve patient outcomes, particularly as patients progress from compensated to decompensated liver disease. Given the dynamics of ongoing research into this area, English-language studies from both animal and human studies were reviewed for inclusion in this review and will be delineated appropriately in forthcoming sections. We aim to guide the reader through the current understanding of the complexities of RAS in liver disease and identify gaps for future study, including the development of novel, pathway-specific therapies.

## 2. Overview of the RAS Pathways

**Figure 1:**
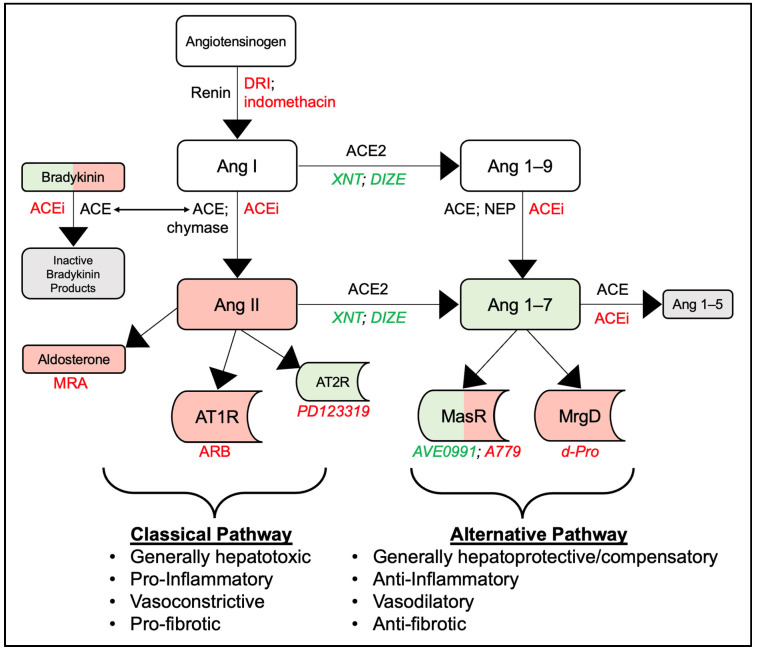
Classical and alternative renin–angiotensin system (RAS) pathways and their hepatotropic effects. Enzymes mediating formation and destruction of products are indicated in black font along the arrow of their respective process. Green font indicates agonist therapies and red font indicates antagonist therapies; experimental-only therapies are indicated by italics. Green shading indicates products that are generally hepatoprotective, red shading indicates products that are generally hepatotoxic, mixed green and red shading indicates products with mixed hepatoprotective and toxic properties, and grey shading indicates inactive products. Abbreviations (in alphabetical order): angiotensin (Ang), angiotensin converting enzyme (ACE), angiotensin converting enzyme inhibitors (ACEi), angiotensin converting enzyme 2 (ACE2), angiotensin receptor blockers (ARB), angiotensin type I receptor (AT1R), angiotensin type II receptor (AT2R), diminazene aceturate (DIZE), direct renin inhibitors (DRI), Mas receptor (MasR), Mas-related-G protein-coupled receptor D (MrgD), mineralocorticoid receptor antagonists (MRA), neprilysin (NEP), xanethenone (XNT).

The origin of RAS is in the liver. All RAS pathways begin with hepatic synthesis of a large protein called angiotensinogen (AGT), which contains 485 amino acids, including a 33-amino-acid signal peptide and 10-amino-acid *N*-terminus. Angiotensinogen is part of a superfamily of serine protease inhibitors (serpins), which include other molecules implicated in liver disease such as alpha-1 antitrypsin and alpha-1 antichymotrypsin [11]. Interestingly, AGT production is upregulated in cirrhosis, despite a global reduction in other protein synthesis, and acts upon hepatic stellate cells (HSCs). This appears to be a positive feedback loop, as activated HSCs secrete further AGT. In contrast, quiescent HSCs in the non-cirrhotic liver do not secrete much AGT [12]. Renin, an enzyme produced by the juxtaglomerular apparatus of the kidney and stimulated by stretch sensors, sodium, and sympathetic tone, then acts upon AGT to form angiotensin I (AngI) [13]. This rate-limiting step allows for further pathway differentiation (Figure 1).

The classical pathway is well established [13,14]. From the AngI precursor, the pulmonary-derived enzymes ACE and chymase independently cleave AngI to form angiotensin II (AngII) [15,16]. Of note, ACE also serves in a separate sequence to cleave bradykinin, which exerts mixed hepatoprotective and hepatotoxic effects given its vasodilatory and anti-fibrotic, yet pro-inflammatory, properties [17,18]. In the main classical pathway cascade, AngII, which also serves as the substrate for aldosterone formation from the adrenal glands, acts on the angiotensin type I receptor (AT1R) and angiotensin type II receptor (AT2R). However, AngII has a stronger affinity for AT1R than for AT2R [13]. Activation of AT1R is generally associated with vascular remodeling, hypertrophy, fibrosis, and release of aldosterone from the adrenal cortex [13]. In contrast, AT2R activation has anti-fibrotic properties to counteract AT1R [19]. The receptor is mostly dormant after embryogenesis in healthy adults, but it is modestly upregulated in liver injury and other conditions of oxidative stress [20]. Nonetheless, the predominance of AngII and its preferential stimulation of AT1R in the classical pathway leads to a pro-inflammatory and pro-fibrinogenic response [20].

Discovery of angiotensin-converting enzyme 2 (ACE2) in 2000 led to characterization of an “alternative” pathway for RAS to exert systemic effects and oppose the classical pathway [5]. This enzyme has multiple targets, leading to shunting of both AngI to angiotensin 1–9 (Ang1–9) and AngII to angiotensin 1–7 (Ang1–7) [21]. Formation of Ang1–7 is also separately promoted by cleavage of Ang1–9 by both ACE and a ubiquitous zinc-dependent endopeptidase called neprilysin (NEP) [15,22]. The ACE enzyme also catalyzes the conversion of Ang1–7 to an inactive molecule called angiotensin 1–5 (Ang1–5) [23]. Active Ang1–7 exerts its effects through two receptors: the Mas receptor (MasR) and the Mas-related-G protein-coupled receptor D (MrgD) [24,25]. The overall downstream effects of this receptor–ligand binding are anti-inflammatory, vasodilatory, and anti-fibrotic, in contradistinction to AngII-mediated effects within the classical pathway. Interestingly, MasR appears to act both locally (intrahepatically) and extrahepatically, whereas MrgD exerts only an extrahepatic influence [13,26]. As a result, the vasodilatory properties of each are somewhat paradoxical in cirrhosis—intrahepatic vasodilatation is beneficial to improve blood flow but dilation of the splanchnic vessels contributes to arterial underfilling and systemic hypotension [27]. Small studies suggest Ang1–7 may have some ACE-inhibiting properties through bradykinin-mediated vasodilation, but further validation is needed [28,29]. Ultimately, the effects of Ang1–7 are potentially pleiotropic but incompletely defined.

The systemic effects of RAS are profound. End effects of the classical RAS pathway include oxidative stress, tissue remodeling, and insulin resistance, which are implicated in multiple diseases including cardiovascular disease, diabetes mellitus, hypertension, heart failure, and hypoxic states [3,4,30]. Furthermore, RAS blockade has been shown to improve COVID-19 outcomes. Hospitalized patients on prior-to-admission RAS-inhibiting medications (i.e., ACE inhibitors and angiotensin receptor blockers) were found to have decreased mortality, suggesting a clinically significant contribution of classical RAS on hypoxia, inflammation, and recoverability [31]. In addition to its relationship to specific disease states, RAS may be influenced by sex-hormone differences. Pre-menopausal women (and post-menopausal women on estrogen replacement therapy) tend to have a more protective RAS balance than men due to an estrogen-driven shift of systemic RAS toward ACE2/Ang1–7/MasR, which facilitates a more favorable metabolic profile [32,33].

Within the liver, the classical RAS pathway has been strongly implicated in dyslipidemia and steatosis formation, including metabolic dysfunction-associated steatotic liver disease (MASLD) [4]. In MASLD, there is an imbalance between the RAS pathways leading to an over-active classical pathway compared to the alternative pathway [4,34]. Clinical prescription of glucagon-like peptide-1 (GLP-1) analogues take advantage of this imbalance by promoting Ang1–7 formation, reducing hepatic steatosis by enhancing carbohydrate metabolism and impairing hepatic gluconeogenesis [4,35]. An additional effect of AngII over-expression is exacerbation of chronic liver-disease-related sarcopenia [36]. While not conclusive, preliminary evidence suggests that increased expression of ACE2 could shunt RAS activity away from AngII and slow muscle depletion [36].

Separate from its systemic effects, RAS also acts locally through paracrine and autocrine signaling on tissue and cellular levels [6]. For example, RAS affects carotid body hemodynamics by regulating sensitivities of chemoreceptors to both acute and chronic hypoxia through its interactions with the dense AngII receptor population in the carotid body [37]. Glaucoma development is also impacted by RAS through its modulation of aqueous humor outflow dynamics, thereby increasing intraocular pressures [38]. Local RAS has even been implicated in carcinogenesis, particularly due to Ang II’s role in angiogenesis, vascular endothelial growth factor (VEGF) activation, and over-activation of macrophage infiltration; RAS inhibitors are currently being studied in an antineoplastic realm [7]. These tissue-specific, direct-acting local systems function independently from the systemic system, though generally mirror the same pathway-specific effects, albeit on a smaller scale. A further review of the local RAS influence on the liver is presented in upcoming sections.

As shown in Figure 1, RAS effects are predicated upon the pathway (classical or alternative) that is preferentially selected, which may change according to different homeostatic conditions, burden of liver disease, and/or the influence of specific RAS modulating therapies. Additional discussion of this modulation and pathway shunting is forthcoming. Nonetheless, it is important to delineate that the final products of the classical and alternative pathways, in general, have contrasting end-organ effects.

## 3. RAS and Liver Fibrosis

Hepatic fibrinogenesis is multifactorial, including contributions from inflammation and reactive oxygen species most often related to chronic injury or rarely fibrosing cholestatic hepatitis, veno-occlusive disease, or biliary obstruction [39,40]. Stellate cells become activated, which ultimately leads to excess collagen deposition in the extracellular matrix [40]. The development of fibrosis though is heavily influenced by local RAS activity. Best described is intrahepatic AngII (from the classical pathway), which executes specific and potent effects on the tissue level. It facilitates transformation of quiescent to active HSCs and ultimately promotes differentiation to myofibroblasts, a process involving harmful reactive oxygen species (ROS) production by nicotinamide adenine dinucleotide phosphate (NADPH) oxidase [12,41]. The result of these cellular events is detrimental, leading to hepatic steatosis and fibrosis in both human and animal cells [42]. Furthermore, activated HSCs support a positive feedback loop in humans, wherein they produce additional AngII and thus activate additional nearby HSCs [12]. Angiotensin II also has specific local effects through stimulating release of endothelin-1, transforming growth factor beta-1 (TGF-β1), interleukin-1, α-smooth muscle actin, and monocyte chemoattractant protein [24]. These molecules have been linked to harmful downstream effects, including smooth muscle proliferation, increased vascular tone, and ultimately increased hepatic vascular resistance [43,44,45]. Specifically, TGF-β1 has been shown in humans to stimulate ROS, which catalyzes further TGF-β1 expression, forming a maladaptive positive feedback loop. As a result, TGF-β1 escalates fibrosis through facilitation of extracellular matrix deposition and hypertrophy of nearby cells [24,46]. In summary, AngII appears to exacerbate hepatic tissue fibrosis in humans both directly and indirectly as part of the local RAS system.

Further evidence for the role of RAS in hepatic fibrogenesis is provided by rodent models. An early study highlighted the endocrine effects of RAS by demonstrating that infusion of systemic AngII exacerbated fibrosis in rats subjected to bile duct ligation, a well-established technique to induce fibrosis and cirrhosis [47]. Other animal models have tried to quantify chronological activation of the classical versus alternative pathways, but the results are contradictory and inconclusive. One animal model utilizing bile duct ligation found that both local and systemic classical pathway components (tissue ACE and plasma AngII) were significantly elevated within one week of the iatrogenic injury [48]. In contrast, alternative pathway components (tissue ACE2 and MasR, plasma Ang1–7), remained normal until 3–4 weeks after the insult, when a significant increase in levels occurred. This time-dependent imbalance suggests that the classical RAS pathway (both locally and systemically) may be swiftly upregulated in fibrosis with a delayed compensatory response through induction of the alternative pathway after the initial insult. However, a similar study model conversely found that serum AngII and Ang1–7 both increased in unison after bile duct ligation [49]. The aforementioned study observed histological fibrosis progression as RAS production continued, suggesting, however, that the Ang1–7 response was inadequate against AngII’s effects. The question of whether alternative pathway activation represents a de novo response to the fibrosis itself versus initial inhibition is unclear and necessitates human study to understand its role in liver disease progression.

## 4. RAS in Cirrhosis, Portal HTN, and the Development of a Hyperdynamic Circulation

### 4.1. RAS in Cirrhosis

While RAS is implicated in fibrosis progression, its role and relative contribution across the spectrum of disease from the non-cirrhotic state to cirrhosis remains incompletely defined. One animal study observing systemic circulation found that the classical pathway was favored in the pre-cirrhotic state with a transition to more alternative pathway shunting in cirrhosis [48]. This is consistent with the theory that upregulation of the alternative pathway is reactionary. However, there is limited evidence to confirm this in humans. In fact, contradictory findings were reported in patients with non-cirrhotic chronic hepatitis C; alternative pathway activation was predominant in earlier fibrotic states [50]. These diverging outcomes highlight the inherent limitations of animal models of cirrhosis, variability within etiologies and degrees of cirrhosis, and suboptimal study design. A prospective study of human patients with longitudinal measurement of systemic RAS activity at set timepoints to capture the journey from pre-cirrhosis to cirrhosis is needed to fully answer the question regarding the evolution of RAS activity.

Measurement of RAS activity in cirrhosis must take into account disease severity and etiology. Cirrhosis in most patients will lead to the development of portal hypertension, defined as a hepatic venous pressure gradient (HVPG) of ≥6 mmHg. Portal hypertension may then increase over time, becoming clinically significant once the HVPG is ≥10 mmHg and/or with the development of decompensations such as ascites, hepatic encephalopathy, or variceal bleeding [51]. In keeping with this paradigm, systemic RAS has been shown to affect progression of compensated to decompensated cirrhosis. There have been only two human studies analyzing this relationship, both of which recruited patients with diverse etiologies of cirrhosis [15,52]. In compensated cirrhosis, both studies demonstrated relatively low systemic levels of both classical and alternative RAS [15,52]. However, there were differences in AngII and Ang1–7 levels. Hartl et al. noted normal AngII and low Ang1–7 levels in patients with compensated cirrhosis, whereas Vilas-Boas et al. found lower levels of AngII (and AngI) and normal Ang1–7 in this population [15,52]. It is important to note a limitation of these results is that neither study controlled for concurrent use of beta blockers, which are known to suppress RAS activity. Nonetheless, it appears that systemic RAS levels are not severely upregulated in compensated cirrhosis, but the relative imbalance between the classical and alternative pathways requires further study given the contradictory findings.

When cirrhosis progresses to a decompensated state, systemic RAS dynamics be-come significantly altered. The two aforementioned studies also recruited patients with decompensated disease [15,52]. A third study investigated serum RAS levels in a population with decompensated hepatitis C cirrhosis, and a fourth study explored this same question in a population listed for liver transplant or receiving a transjugular intrahepatic portosystemic shunt (TIPS) [50,53]. Across all four studies, there was increased activation of AngII and Ang1–7 in the decompensated state [15,50,52,53]. There was consistent evidence for elevated renin and AngI levels; Hartl et al. also observed high aldosterone levels in patients with decompensated cirrhosis [15]. The majority of studies provided strong evidence for ACE activation, although Casey et al. instead noted elevated ACE2 levels with progression of liver disease [53]. Although the combined sample size of patients in these studies is small (*n* = 142), there appears to be more consistent evidence for RAS activation in decompensated cirrhosis compared to the compensated state.

The effect of progressive liver disease on alternative pathway and Ang1–7 levels remains unclear given the discrepant findings. Based on the limited aforementioned studies, there is some evidence that Ang1–7 levels are increased in the pre-cirrhotic state, then decline in compensated cirrhosis, before rising again once a patient develops decompensation [15,50,52]. One possibility is variation in study populations. The only study measuring systemic RAS levels in non-cirrhotic liver disease studied patients with hepatitis C, making it difficult to exclude viral-mediated injury rather than the inherent liver disease itself [50]. Ongoing hepatic injury, for example through active viral infection or heavy alcohol use, could independently stimulate systemic RAS. Unfortunately, there is a lack of studies comparing RAS activation between various liver disease etiologies (i.e., alcohol, metabolic, viral, biliary, etc.) to assess the effect of this potential confounder. Prospective study measuring RAS activity across the spectrum of liver disease with subgroup analysis by cirrhosis etiology is needed.

When considering disease severity, Ang1–7, in particular, has significant clinical relevance. Levels of Ang1–7 correlate with the acute phase reactant interleukin-6 (IL-6) in humans and also with the Model for End-Stage Liver Disease (MELD) score [15,50]. Both IL-6 and MELD are independently correlated with disease severity and increased morbidity and mortality [54,55]. Given that IL-6 is pro-inflammatory and the MELD score is a well-validated surrogate measure of liver disease severity, increased expression of Ang1–7 may represent a compensatory response to inflammation [56]. Its efficacy, however, remains unknown.

As previously mentioned, RAS exerts both systemic and local effects in liver disease. While its systemic effects have been described, local RAS activation is context-dependent in humans. In compensated cirrhosis, local RAS appears hyperactive, with some attenuation in decompensated cirrhosis (albeit still relatively active) [57,58]. These local RAS components include classical (ACE, chymase) pathway enzymes as well as alternative pathways enzymes (ACE2, NEP). While the existence of a relevant imbalance between these competing entities is unproven on a local level, clinical observation is supportive [15]. Many patients with cirrhosis will have development and progression of portal hypertension over time, often related to increasing fibrinogenesis, which is modulated by the effects of AngII. Interestingly, the effects of RAS on a local versus systemic level are different depending on disease severity. As cirrhosis progresses from compensated to decompensated, the high-concentration RAS shifts from local to systemic pools, but the mechanism is unknown [15]. Further study to investigate this proposed pathophysiologic explanation, with the inclusion of healthy controls and patients across the spectrum of liver disease, is required.

### 4.2. RAS in Portal Hypertension, the Hyperdynamic Circulation, and Hepatocellular Carcinoma

The development and progression of portal hypertension in cirrhosis leads to overt clinical manifestations such as ascites, hepatic encephalopathy, and the presence of varices (+/−bleeding). This phenomenon is driven primarily by two processes: splanchnic vasodilation causing increased portal venous inflow, and increased hepatic vascular resistance related to fibrosis and intrahepatic vasoconstriction [59,60]. In early cirrhosis, the body can compensate for these physiologic abnormalities by augmenting cardiac output alone to maintain normal arterial blood pressure [52,61,62]. As fibrosis and splanchnic vasodilation progress, however, increased cardiac output cannot maintain effective arterial blood volume (EABV). In turn, the body attempts to increase EABV by increasing AngII and aldosterone. The end effect of this upregulation is promotion of renal sodium and water retention through reabsorption in principal cells and aquaporins in the collecting ducts. Unfortunately, this process exacerbates ascites formation due to portal vein backup and impaired lymphatic drainage in the environment of decreased oncotic pressure and increased vascular permeability [63]. Additionally, the body attempts to promote vasoconstriction to counteract the low EABV by preferential activation of the classical RAS pathway through AngII (via AT1R), increasing sympathetic tone (mediated by epinephrine and norepinephrine) and expression of antidiuretic hormone, endothelin, thromboxane, and leukotrienes [41,45,64,65,66]. Ultimately, however, these counteracting mechanisms cannot overcome massive splanchnic vasodilation. Furthermore, both animal and human studies have shown that the splanchnic vessels themselves contribute to low EABV. The splanchnic vasculature is hyporesponsive to vasoconstrictive efforts and hyperresponsive to vasodilatory factors such as nitric oxide [66,67,68]. Ultimately, a hyperdynamic but inefficient circulation results in systemic hypotension and portends significant morbidity and mortality [57,69].

In addition to its contribution to the hyperdynamic circulation, RAS plays a role in exacerbating portal hypertension. The alternative pathway generally promotes vasodilation; intrahepatically this is beneficial to reduce vascular resistance but is maladaptive when affecting the splanchnic vasculature. On a local level, the splanchnic vessels exhibit elevated levels of ACE2 and Ang1–7, as shown in both human and animal models of cirrhosis [27]. These alternative pathway components induce harmful and potent nitric oxide release in the splanchnic vessels and lead to decreased total peripheral resistance, splanchnic pooling, and hypotension in cirrhosis [70]. Moreover, the alternative pathway is preferentially active compared to the classical pathway in the splanchnic bed; this imbalance becomes significant, such that the ratio of Ang1–7/AngII concentrations has even been found to correlate with cardiac output in decompensated cirrhosis in one human study [52]. Thus, while the alternative pathway has traditionally been considered hepatoprotective given its robust anti-inflammatory and anti-fibrotic properties, its negative effects in splanchnic vasculature highlight the complexity of the RAS system when evaluating systemic and local effects.

Given the significant role that the alternative RAS pathway plays in the development and progression of portal hypertension, there is increasing interest in defining the specific roles of MasR and MrgD. However, data remain sparse. In a mixed human patient and rat model of cirrhosis, MasR was found to exert local hepatoprotective properties through facilitation of the vasodilatory, anti-fibrotic, and anti-inflammatory actions of Ang1–7 [27]. These mechanisms decrease portal resistance and thus portal hypertension. In contrast to MasR, other studies investigating MrgD found that it was not significantly active within the liver rats with cirrhosis [41,71]. The presence and actions of these receptors shifts when considering the splanchnic circulation. Both MrgD and MasR are active within this vascular bed and enable profound vasodilatory effects, promoting hyper-dynamic circulation in the cirrhotic state [27]. Thus, the current knowledge supports that the primary contribution of MrgD in cirrhosis may be in its harmful splanchnic vasodilation. Corroborating human studies, however, are required to confirm these effects. Compared to MrgD, MasR appears to have a more complex physiologic effect and promotes both beneficial and harmful effects in cirrhosis through reduction in portal resistance and increasing splanchnic circulation, respectively (Figure 1). Overall, the few aforementioned studies depict well-defined roles of MasR and MrgD but are limited in their generalizability by low sample sizes and need for reproducibility. Nonetheless, these preliminary findings reveal potential future therapeutic targets for treating portal hypertension.

Portal hypertension contributes to negative outcomes in cirrhosis and is exacerbated by multiple components of RAS. As the catalyst for the conversion of angiotensinogen to AngI, renin has been incriminated in maladaptive liver hemodynamics. Bosch et al. found that plasma renin levels directly correlated with wedged hepatic vein pressures [57,72]. Numerous current studies continue to associate renin with poor outcomes. In large human observational studies of cirrhosis, plasma renin levels have been found as independent risk factors for the first decompensation event, hyponatremia, hypotension, and mortality [57,69,73,74]. Renin levels, like Ang1–7 levels, even correlated with MELD score in a small human study [57]. While plasma renin levels are not routinely measured in the clinical setting, these recent findings present an avenue for improving clinical care. However, their utility is likely confounded by the frequent use of loop diuretics, mineralocorticoid receptor antagonists, ACEi, and ARBs in this population (whether specifically for the underlying liver disease or non-hepatic co-morbidities) [73,75]. Clinicians would also face challenges in interpreting one-time plasma renin levels of individual patients with cirrhosis, particularly considering the predicted difficulty of standardizing reference ranges. Instead, the greatest utility of plasma renin levels may be for prognostic purposes to identify patients at high risk for disease and portal hypertension progression. Prospective studies are needed to confirm this potential application.

**Figure 2:**
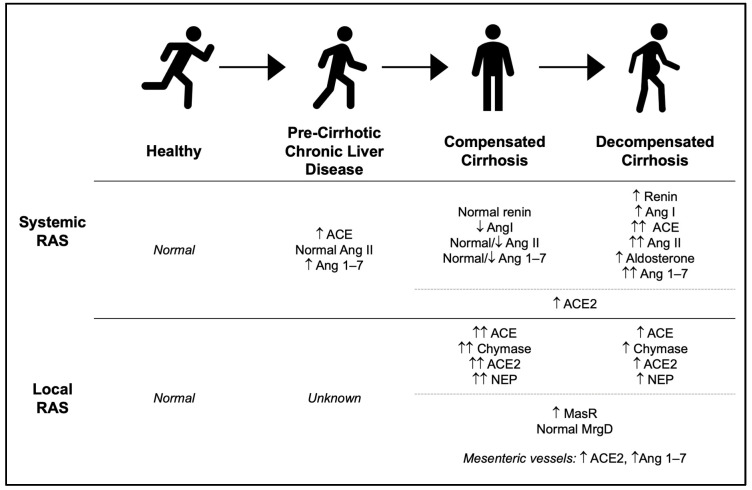
Systemic and local RAS activity in humans across the spectrum of liver disease. Alterations in the balance between classical and alternative pathway effects begins with the development of cirrhosis and becomes more pronounced as decompensation occurs.

Overall, the development of cirrhosis and portal hypertension is associated with alterations in RAS activity, both on a systemic and local level (Figure 2). Traditional understanding has revolved around the development of decompensated liver disease leading to upregulation of the classical pathway via AngII and aldosterone. However, there is emerging evidence that the alternative pathway is also active and its effects may further exacerbate systemic hypotension through splanchnic vasodilation. Further research into the balance between the classical and alternative pathways is needed, particularly with respect to the pre-cirrhotic state and in compensated cirrhosis, where there are contrasting findings. Additional characterization of this RAS dysregulation is needed to highlight pretransplant interventions, which may reduce the morbidity and mortality associated with portal hypertension-related events.

The effects of RAS also influence the development of hepatocellular carcinoma (HCC), a common complication in cirrhosis with a rising worldwide incidence [76]. The classical RAS pathway appears to be involved in carcinogenesis, with AngII promoting cellular proliferation, angiogenesis, vascular endothelial growth factor (VEGF) activation, and over-activation of macrophage infiltration [6,7]. Interestingly, upregulation of ACE2 (shifting the balance of RAS activation to the alternative pathway and thus towards Ang1–7) has been shown to improve prognosis in HCC in mice [77]. There may be additional benefits of ACE2 activation, as it inhibits the Warburg effect, the phenomenon in which cancer cells preferentially consume and monopolize glucose [77]. Furthermore, experimental animal models have shown that institution of RAS blockade with ACEi/ARBs can decrease progression and development of new HCC lesions [78,79,80]. Purposeful RAS modulation as a therapeutic or adjuvant option in the treatment of HCC in humans represents an untapped novel target, particularly in an era of personalized medicine.

## 5. RAS and the Kidneys in Liver Disease

Renal dysfunction in patients with liver disease, especially cirrhosis, is common and associated with poor outcomes in the absence of transplant [81,82]. An early study identified decreased renal perfusion as a trigger for increased plasma renin levels in cirrhosis-related kidney injury [75]. Over the past several decades, further research has revealed that both renin and AngII are elevated in cirrhosis-related renal dysfunction. These two components initially cause vasoconstriction, increase blood pressure, and improve the glomerular filtration rate (GFR), the sum of which is initially beneficial [61,83]. However, as cirrhosis and portal hypertension progress, excessive splanchnic vasodilation and diminished cardiac output lead to decreased EABV. The compensatory elevation in renin and AngII levels becomes maladaptive, causing further arterial renal vasoconstriction and decreasing GFR, eventually leading to hepatorenal syndrome (HRS) in severe cases [83,84,85]. Simultaneously, AngII facilitates renal fibrosis through release of fibrogenic and inflammatory molecules (i.e., TGF-β1, IL-6, and metalloproteinases), increasing extracellular matrix production, and promoting cellular proliferation [86]. Combined with the renin-induced vascular dysfunction, renal fibrinogenesis contributes to a further GFR decrease [86].

While contributions from renin and AngII (classical pathway) are well-defined in cirrhosis-related renal dysfunction, the role of the alternative pathway is less clear. In advanced cirrhosis, Ang1–7 opposes the effects of AngII by promoting afferent renal artery vasodilatation. This increases GFR, although specific mechanisms driving this process remain undefined [61,70,87]. The actions of Ang1–7 on sodium and water balance are incompletely settled. Some studies have found Ang1–7 to act upon the proximal nephron to promote natriuresis and diuresis, while other studies have found Ang1–7 to increase water resorption in the distal nephron [88,89]. Regardless of the mechanism, the alternative pathway appears to generally have renoprotective effects. In critically ill patients, higher urine ACE2 levels have been associated with a lower risk of severe acute kidney injury (AKI) [90]. One interpretation of these results is that a reduced ability to upregulate ACE2 expression may contribute to severe AKI in critical illness, although further study is required to confirm this. Overall, the alternative RAS pathway may promote renal perfusion and have renoprotective effects, but the existing evidence base remains small.

## 6. Modulation of Classical RAS

The interplay of classical RAS pathway modulators, primarily ACEi and ARBs, and their effects in liver disease, comprise an emerging area of research interest. Their study is aided by practical benefits, including widespread use for common comorbidities such as hypertension and diabetes [4]. The predominant pharmacologic effect of ACEi administration is inhibition of AngII formation from AngI. However, ACEi also exerts a modest inhibitory effect on both formation and breakdown of Ang1–7, although the inhibition of break-down is favored and results in a net increase in Ang1–7 levels (Figure 1) [14,23]. The mechanism of ARBs, on the other hand, is further downstream. These enzymes inhibit the classical pathway receptor AT1R (but not AT2R), the receptor through which AngII exerts most of its actions. Additionally, ARBs indirectly increase Ang1–7 concentration via the increase in its substrate AngII, which is then converted to Ang1–7 by ACE2 (Figure 1) [91,92]. Modulation of AT2R is less common; a single agent called PD123319 has been studied but demonstrates poor selectivity and has not received wide adaptation for clinical use [25,47]. Although ACEi and ARBs exert secondary effects on the alternative pathway, they primarily modulate the classical pathway. Their effects have been studied within several aspects of liver disease, including fibrosis, portal hypertension, and renal dysfunction.

### 6.1. RAS Inhibition in Liver Fibrosis

In most human studies, RASi (ACEi or ARB use) has correlated with both histological and score-based improvement of fibrosis in patients with chronic liver disease or cirrhosis [93,94,95]. In one meta-analysis of seven human studies, RASi was associated with decreased serum markers of fibrosis, including TGF-β1, collagen I and IV, and matrix met-alloproteinase-2 [93]. Conversely, other human studies have not found an association between RASi and liver fibrosis [96,97]. These discrepancies could potentially be explained by differing results based on population. In a high-powered, intention-to-treat case–control study looking at RASi in MASLD, RASi was not effective at prevention and progression of MASLD overall, although it was effective in subgroup analysis of obese subjects within a 5–6-year timeframe [98]. Overall, the preponderance of evidence supports an anti-fibrotic effect of RASi in liver disease. In the presence of non-decompensated disease and absence of other contraindications, initiation or continuation of RASi for other comorbid conditions (i.e., hypertension, heart failure, etc.) appears reasonable.

### 6.2. RAS Inhibition in Cirrhotic Portal Hypertension

Clinical guidelines have cautioned against the use of RASi in cirrhosis, especially with ascites [99,100]. These recommendations were informed by prior studies, which showed a decrease in mean arterial pressure and higher rates of ESRD with RASi use in this population [65,101,102,103]. As previously discussed, AngII-mediated vasoconstriction causes an initial increase in GFR in early cirrhosis, but GFR decreases with continued vasoconstriction in advanced cirrhosis. When RASi is added, older studies demonstrate that dilation of renal efferent arterioles decreases GFR and exacerbates hypoperfusion in cirrhosis [14]. However, the discovery of RAS-mediated splanchnic vasodilation has been coupled with conflicting results for RASi efficacy in reducing portal pressures. Presently, ARBs have been studied more often than ACE with regard to effects on portal pressure and appear to be more potent in observational human studies, although direct comparison is lacking (see Table 1) [65,102,104,105,106,107,108]. An explanation for this could be related to ARB-mediated direct blockade of AT1R, allowing for increased conversion of AngII to Ang1–7 via ACE2 and thereby shunting toward the alternative pathway. Yet this benefit is partially muted by the induction of feared side effects in some patients, including hypotension and renal impairment [71,102]. One small study reported that 22% of patients receiving irbesartan developed severe symptomatic hypotension within two days [102]. Importantly, the patients who were intolerant to ARBs were typically those who had ascites or a higher plasma renin, indicating pre-existing decompensation [102]. Other studies of ARB use have failed to show an effect on portal pressure, but these are fewer than the supporting studies [103,109,110,111,112,113]. In contrast, research on ACEi in portal hypertension is too sparse to impact clinical practice (Table 1) [114,115,116]. A large, multicenter study evaluating RASi ability to mitigate portal hypertension is needed with subgroup analyses of compensation status and RASi type.

Based on available evidence, RASi appears relatively safe but only modestly effective in decreasing portal pressure in compensated cirrhosis. In decompensated cirrhosis, however, there are numerous studies reporting a high proportion of adverse outcomes with RASi. These agents are associated with the development of hypotension and renal dysfunction, especially in patients with ascites [102,103,109,110]. Given significant underlying splanchnic and low EABV, patients with decompensated cirrhosis are intolerant to further reductions in blood pressure or GFR [65,117]. Safety aside, RASi may merely be less effective in decreasing portal hypertension in the decompensated state. As previously discussed, advanced cirrhosis is characterized by a profound activation of both RAS-dependent and RAS-independent mechanisms (including endothelin and sympathetic systemic) to raise blood pressure. Thus, RASi may not be able to overcome RAS-independent factors [64,65,118]. Until studies are replicated with larger sample sizes in decompensated cirrhosis, which may be difficult to conduct given ethical concerns regarding increased risk of adverse events, RASi is likely best limited to earlier stages of liver disease. Clinical judgement should guide its continued use after decompensation and close monitoring is required.

**Table 1:** Human studies evaluating the effects of ACEi and ARBs on liver fibrosis and portal hypertension in liver disease [65,98,102,103,104,105,106,107,108,109,110,111,112,113,114,115,116,119,120,121,122]. Italicized studies indicate meta-analyses. The effects of ARBs are more potent and better studied than ACEi in compensated liver disease. Research to routinely support the use of RASi therapy in decompensated disease is lacking.

**ACEi…**		
**Improves fibrosis?**	**Decreases portal pressure?**	**Has no effect on portal pressure?**
Zhu 2016 Kim 2021	Svoboda 1992 Baik 2003	Tsai 1996
**ARB…**		
**Improves fibrosis?**	**Decreases portal pressure?**	**Has no effect on portal pressure?**
Yokohama 2004 Sookoian 2005 Kim 2012	Schneider 1999 Schepke 2001 * Castaño 2003 De 2003 Debernardi-Vernon 2007 Tandon 2010 Hidaka 2011	Gonzalez-Abraldes 2001 * Tripathi 2004 * Heim 2007 * Schepke 2008 Agasti 2013 Kim 2014

* Medication-related adverse events include hypotension or renal dysfunction.

### 6.3. RAS Inhibition and the Kidneys in Liver Disease

Inhibition of RAS has been thought to have differential effects on renal function depending on the stage of liver disease. Large cohort studies have begun to signal that RASi may provide overall renoprotective effects in patients without decompensated cirrhosis. In a recent retrospective cohort study of MASLD patients, which included patients with both compensated cirrhosis and non-cirrhosis, ACEi use was associated with lower rates of adverse liver-related events (defined as decompensating events and/or development of hepatocellular cancer). The same study also demonstrated efficacy of ACEi in those with chronic kidney disease (CKD) [123]. These findings are echoed by other large, well-executed human studies which similarly showed that RASi correlates with improved outcomes and fewer liver-related events in compensated cirrhosis, while there was no association with ESRD [101,124]. Thus, RASi use in the non-cirrhotic and compensated cirrhotic states appears to have an overall beneficial effect. It is important to note, however, that these studies were not specifically designed to demonstrate reduction in renal dysfunction and there is a lack of evidence to support sole initiation of RASi for this purpose.

In patients with decompensated cirrhosis, the effects of RASi on renal outcomes are more muddled. Multiple studies have shown that patients with decompensated cirrhosis with ascites who take RASi experience higher rates of ESRD, and this risk has led to the widespread avoidance of RASi in this population [99,101,102,103]. In contrast, a recent single-center retrospective study found that there were decreased rates of severe AKI and need for hemodialysis in patients on RASi therapies [125]. The study population included patients taking RASi for a comorbid indication prior to decompensation. Given the study design and small number of patients within the RASi use group (*n* = 41), unmeasured confounders and statistical power are limitations. Nonetheless, these intriguing results suggest that universal discontinuation of RASi after decompensation may not be necessary. Whether these results can be replicated in larger, multicenter studies and whether certain patient subgroups derive particular benefits from RASi continuation after decompensation are important to understand before clinicians routinely continue or institute RASi in decompensated cirrhosis.

### 6.4. Differential Effects of ACEi and ARBs in Liver Disease

Characterization of the relative effects of ACEi versus ARBs in liver disease is increasing in interest. Prior studies have not explicitly differentiated between the classes when looking at overall RASi effect on outcomes such as mortality in cirrhosis [124]. In rats, a few studies have shown that ARBs attenuate liver fibrosis through reduction in hepatic stellate cell activation and collagen deposition, decreased oxidative stress, and lower TGF-β1 levels in superior fashion to ACEi [126,127]. In human studies, however, strong preference for ARBs is not yet well supported.

Losartan has been shown to attenuate fibrosis in patients with chronic hepatitis C and alcohol-associated liver disease, as well as to attenuate steatosis in MASH and MASLD, although a comparison to ACEi was absent (Table 1) [121,122,128,129]. In a small human study, all seven MASH patients had favorable transaminases, plasma TGF-β1, and ferritin levels after losartan administration; histological fibrosis improved in 4/7 patients [120]. In contrast, other studies have conversely found no improvement in surrogate markers of fibrosis with use of ARBs [130,131]. A comparison between ACEi and ARBs across multiple etiology of liver disease was recently reported in a large Swedish database. The authors found that the use of ACEi was associated with lower mortality in alcohol-associated liver disease and less morbidity in viral liver disease, while ARB use lacked similar benefit [132]. Despite the large sample size, unmeasured confounders may have been present and adverse event data were not reported. In large observational studies of MASLD patients, ACEis have similarly been shown to be more effective than ARBs in slowing fibrosis progression, decreasing hepatic decompensation, reducing rates of primary liver cancer, and improving mortality [98,123,133]. While this is intriguing, prospective study and, ideally, randomized clinical trials, are necessary to confirm preferential benefits of ACEi over ARBs. Moreover, the mechanism for clinical benefit is unclear. One theory is that ACEi use leads to accumulation of bradykinin, which has been shown to have hepatoprotective effects [17,98]. This may be through bradykinin’s reduction in hepatic fibrosis by inhibiting deposition of fibrogenic components such as pro-collagen and TGF-β1, as shown in rat models of chronic liver injury [17]. However, bradykinin may also have hepatotoxic effects, and thus its upregulation as the sole explanation for ACEi superiority is not definitive [127]. Overall, however, it remains unclear whether ACEi or ARB use is superior with respect to liver-related outcomes in patients with liver disease and cirrhosis. Given a higher prevalence of comorbid metabolic conditions in patients with steatotic liver disease, further studies will also need to take this into consideration when selecting patient populations and appropriately powering outcomes.

### 6.5. Other RAS Modulators

Outside of ACEi and ARBs, other RASi therapies have been studied in liver disease. Indomethacin has been shown to block renin in alcohol-associated liver disease, although its mechanism is unknown [134]. Non-selective beta blockers (NSBBs), which were first reported to decrease variceal bleeding in 1981, are well-established agents utilized to decrease portal hypertension through splanchnic vasoconstriction [135]. With regard to RAS, NSBBs are inhibitory overall through reduction in renin and AngI expression, although they do not necessarily promote upregulation of the alternative pathway given their neutral effect on the ratio of Ang1–7/AngII [136]. Some studies have compared NSBBs to ARBs with regard to their effects on portal hypertension; a narrow majority have found that ARBs offer no discernible advantage when used alone nor an additional benefit when used in combination with NSBBs [65,103,113]. The latter finding requires additional investigation, however, as RASi has been shown to reduce portal pressures, and patients in other large observational studies may have been taking concomitant NSBB therapy, given high prevalence of NSBB use in cirrhosis in general [124,125].

Mineralocorticoid receptor antagonists (MRAs), such as spironolactone or eplerenone, have also been studied in combination with NSBB and provide additional portal pressure reduction [137]. Aldosterone is a classical pathway component and contributes to salt and water retention at the distal tubule of the nephron [63,138]. By inhibiting aldosterone, MRAs are natriuretic. The result of this is an increase in plasma renin, which is better established in heart failure but has also been documented in cirrhosis [139,140]. In cirrhosis, however, there is a lack of clarity about the effect of this excess renin and whether there is preferential selection for the classical RAS pathway or shunting to the alternative pathway. In general, MRAs are commonly utilized given their tolerability and success in treating ascites in combination with loop diuretics, but current studies quantifying hemodynamic effects of MRAs in cirrhosis are sparse [65,137,141]. Given the complexity of RAS and discovery of the alternative pathway since the original research was published on MRA use in cirrhosis, new research to revisit the effects of MRA on cirrhosis physiology is needed.

Midodrine is an alpha-1 receptor agonist and another RAS modulator. Outside of its use in HRS, the addition of midodrine to octreotide has favorable effects on the hyperdynamic cirrhotic circulation and provides a buffer against renal dysfunction related to octreotide monotherapy in patients with ascites [142]. Both agents decrease plasma renin levels, but midodrine is more potent and has the advantage of increasing GFR compared to a decrease in GFR with octreotide alone [142,143,144]. When compared to albumin infusion, midodrine was non-inferior across multiple outcomes, including overall mortality, and in preventing paracentesis-induced circulatory dysfunction, midodrine was non-inferior in a recent meta-analysis [144]. Given that midodrine has been associated with lower levels of renin and aldosterone, it could be considered an inhibitor of the classical RAS pathway. Its mechanism of action and effect on the alternative pathway, if any, remain unknown. As a result, it is also possible that midodrine has no direct effects on RAS and rather its association with decreased renin and aldosterone are downstream effects of improved hemodynamics. Future work would be best directed toward understanding if midodrine has RAS-specific effects and confirming the benefit of chronic midodrine use in improving cirrhosis-related outcomes.

Lastly, vaptans are vasopressin (anti-diuretic hormone) receptor antagonists used in refractory hyponatremia to facilitate urinary free water excretion. By this same mechanism, vaptans have also been found to be effective at controlling ascites without affecting intravascular volume nor RAS, including unchanged renin and aldosterone levels [145,146]. Despite these benefits, vaptans have traditionally been discouraged in liver disease given concerns surrounding hepatotoxicity in patients with both liver and non-liver disease [147,148,149]. Given their utility in refractory hyponatremia, however, newer studies have revisited vaptan use and refuted their rigid avoidance for short-term use [150,151,152]. These studies have found no significant vaptan-related safety concerns, suggesting a need for reconsideration of stringent anti-vaptan language in current clinical guidelines.

## 7. Modulation of Alternative RAS

Since the discovery of ACE2 and MrgD, observational and experimental studies have attempted to elucidate their importance in liver disease [5,25]. These studies have predominantly utilized animal models but show high potential for future clinical relevance in patients with liver disease. As previously stated, ACEi and ARBs have alternative RAS-modulating properties (in addition to their primary role as classical RAS modulators) through increasing Ang1–7 concentrations [14,23]. In understanding the alternative pathway, novel interventions targeting ACE, Ang1–7, MasR, and MrgD have been investigated.

Modulation of ACE2 and Ang1–7 is an area of active research. Although administration of Ang1–7 has not yet been trialed in humans, intravenous infusion in rodent studies is associated with decreased hepatic resistance and attenuated fibrosis [27,50]. More recently, an oral Ang1–7 formulation developed in rodents decreased emphysematous changes and exhibited cardioprotective properties by mitigating cardiac remodeling [153,154]. Theoretically, therapies augmenting ACE2, the alternative pathway enzyme that degrades AngII and upregulates Ang1–7, should have similar outcomes. Recombinant human ACE2 (rhACE2) is one such drug; rhACE2 is successfully making its way into human trials for conditions such as acute respiratory distress syndrome (ARDS) and heart failure, but its hepatic effects are ill defined given scarcity of studies [30,155,156]. Only one mouse study has investigated rhACE2, although it showed a beneficial effect in reducing hepatic fibrosis [155]. Other promising agents are the “ACE2 activator” therapies xanethenone (XNT) and diminazene aceturate (DIZE). In animals, XNT activates ACE2, upregulates Ang1–7, inhibits AngII, and decreases cardiac and renal fibrosis, including those with ARDS [157,158]. The liver-related effects of XNT have not yet been studied in either animals or humans. The other ACE2 activator, DIZE, decreases liver fibrosis in mice, although its exact mechanism is unclear [159]. Administration of Ang1–7, rhACE2, and ACE2 activator drugs (XNT and DIZE) have local, tissue-level hepatoprotective effects in rodents and are promising in other organ systems, but their systemic effects in human liver disease are unknown and further animal studies are required to confirm their efficacy and safety. Specifically, augmentation of ACE2 could theoretically lead to harmful hypotension in liver disease. To avoid this risk, Mak et al. trialed a promising adeno-associated viral vector in mice. The authors found that hepatic fibrosis was impaired in all etiologies tested (cholestatic biliary fibrosis, alcohol-related fibrosis, and MASLD); the therapy was reportedly safe, although it is unclear if blood pressure or renal function were monitored [160].

Another alternative RAS pathway target for potential modulation is neprilysin, which cleaves Ang1–9 to form Ang1–7. Inhibition of neprilysin, combined with angiotensin receptor blockade (ARNi), has been used to treat mild-to-moderate heart failure [22]. Experience with ARNi use in cirrhosis, however, is limited to animal studies. However, AR-Nis were found to decrease portal pressure, even compared to ARBs alone in one of two studies [161,162]. The favorable effect of neprilysin inhibition may initially seem counterintuitive, since neprilysin promotes Ang1–7 formation in the alternative pathway (Figure 1). However, neprilysin also has pleiotropic effects, including elimination of brain natriuretic peptide (BNP) and other natriuretic peptides, which are profoundly diuretic, vasodilatory, and anti-fibrotic [22]. These beneficial natriuretic peptides are secreted by the heart in response to increased blood pressure, volume, and hypertrophy, and they act to oppose these effects [22]. Neprilysin inhibitors alone may not be beneficial given their mixed effects, but combination therapy with ARBs effectively blocks the activity of AngII while continuing to allow the production of Ang1–7 via ACE2. Due to redundancy in Ang1–7 formation in RAS (i.e., fed by two pathways as seen in Figure 1), Ang1–7 formation is preserved [163]. Unfortunately, enthusiasm for further pursuit of ARNi use in cirrhosis, particularly in the decompensated state, may be tempered by the increased risk of developing hypotension [161,162]. Paralleling its approved use in early heart failure as opposed to advanced heart failure, it is possible ARNi use could have benefits in earlier stages of liver disease, but human studies are required to confirm this hypothesis.

Downstream in the alternative pathway, researchers have investigated both the agonism and antagonism of MasR, a receptor for Ang1–7. One such agent is AVE 0991, an oral MasR agonist which in animal models of cirrhosis decreases α-smooth muscle actin and hydroxyproline production by hepatic stellate cells [50,164]. Opposing these effects is A779, an intravenous MasR antagonist found to exacerbate liver fibrosis and increase hydroxyproline and TGF-β1 [49]. Thus, it appears that MasR agonism in animals has antifibrotic effects in the liver. In contrast, the effects of MasR on portal hypertension are mixed (Table 2). Some studies report that MasR agonism decreases portal pressure in cirrhosis [26,164], while others report that antagonism does the same [27,71]. These contrary results can be reconciled by understanding the twofold role of MasR in liver disease. Grace et al. found that MasR antagonism resulted in a reduction in maladaptive splanchnic vasodilation yet concurrently increased hepatic resistance [27]. However, the true effect of MasR on portal hypertension is further muddled by another study that did not demonstrate a reduction in splanchnic vasodilation with MasR blockade [26]. Further study is required to better characterize MasR’s systemic role in liver disease, its potential role alongside ARBs or ACEi, and its effects with differing etiologies of liver disease (viral, alcohol-associated, steatotic, etc.).

The current knowledge surrounding MrgD modulation is more straightforward than that of MasR. An MrgD blocker called D-Pro has been trialed in rodents but not yet in humans. In cirrhotic rats, continuous infusion of D-Pro restored normal splanchnic vessel resistance and reduced portal pressures by 33% (Table 2) [26,71]. These early findings are encouraging, and future studies on pharmacokinetics, reproducibility, and clinical application should be pursued. Given the clearer effects of MrgD compared to MasR, MrgD may be a more prudent target in the future for improved consistency and lesser likelihood of unwanted effects. Nonetheless, agents targeting MasR and MrgD require investigation in human populations before firm conclusions can be drawn about the clinical effects of agonism and/or antagonism.

## 8. Conclusions

The renin–angiotensin system (RAS) in liver disease is complex and its numerous roles are still incompletely understood. However, significant progress has been made over the past few decades, including defining the existence of the alternative pathway as a counterbalance to the classical pathway. In particular, interplay between the classical and alternative pathways remains an area of ongoing research and dysregulation may play a role in the progression of liver disease. Moreover, the ongoing characterization of local RAS effects alongside systemic RAS effects has shed light on understanding some of the drivers of hepatic fibrosis and development of portal hypertension. Nonetheless, an important limitation of the current literature is the inclusion of varying etiologies of liver disease (i.e., alcohol, MASH, viral hepatitis, autoimmune, etc.) in study populations. This represents an important potential confounder, and the impact of disease-specific influences on RAS activation and pathway balance (independent of hepatic fibrosis or portal hypertension) represents an opportunity for further research. Finally, research on modulation of both classical and alternative RAS pathways with both established and novel therapies continues to provide clinically relevant insights. Future directions regarding RAS in patients with liver disease should include a focus on targeted systemic therapeutic strategies (such as alternative pathway agonism rather than just classical pathway antagonism), tissue-specific therapies that utilize new discoveries of local RAS to avoid systemic side effects, and trials of therapies with proven benefits in extrahepatic disease.

## Figures and Tables

**Table 2:** Observed effects of alternative pathway receptors on fibrosis and portal pressure in animal studies [26,27,49,50,71,164]. There are no corresponding human studies.

**MasR…**			
**Improves fibrosis?**	**Decreases portal pressure?**	**Increases portal pressure?**	**Has no effect on** **portal pressure?**
Pereira 2007 Lubel 2009	Klein 2015	Grace 2013 Gunarathne 2019	Gunarathne 2022
**MrgD…**			
**Improves fibrosis?**	**Decreases portal pressure?**	**Increases portal pressure?**	**Has no effect on** **portal pressure?**
No studies	No studies	Gunarathne 2019 Gunarathne 2022	No studies
